# Evaluation of an Acute Osmotic Stress in European Sea Bass via Skin Mucus Biomarkers

**DOI:** 10.3390/ani10091546

**Published:** 2020-09-01

**Authors:** Borja Ordóñez-Grande, Pedro M. Guerreiro, Ignasi Sanahuja, Laura Fernández-Alacid, Antoni Ibarz

**Affiliations:** 1 Department of Cell Biology, Physiology and Immunology, University of Barcelona (UB), 08028 Barcelona, Spain; b.ordonez@ub.edu (B.O.-G.); isanahuja@ub.edu (I.S.); tibarz@ub.edu (A.I.); 2 CCMAR—Centre for Marine Sciences, University of Algarve, 8005-139 Faro, Portugal; pmgg@ualg.pt

**Keywords:** *Dicentrarchus labrax*, hypersalinity, hyposalinity, mucus exudation, osmolality

## Abstract

**Simple Summary:**

Skin mucus biomarkers have become relevant indicators for studying fish physiological status and welfare. Here, we evaluated them in terms of the acute osmotic response of the sea bass. Change of mucus volume exuded and main stress-related metabolites explain the putative energy loss implied in a hyper/hypo-osmotic response. We demonstrated that skin mucus is a valuable tool, comparable to classical blood markers, for evaluating sea bass response to acute salinity challenges as well as some other potentially stressful situations. This technique will allow ecologists, physiologists, and aquafarmers to monitor fish welfare and to analyse endangered migrating species without affecting their vulnerable populations.

**Abstract:**

European sea bass is a marine teleost which can inhabit a broad range of environmental salinities. So far, no research has studied the physiological response of this fish to salinity challenges using modifications in skin mucus as a potential biological matrix. Here, we used a skin mucus sampling technique to evaluate the response of sea bass to several acute osmotic challenges (for 3 h) from seawater (35‰) to two hypoosmotic environments, diluted brackish water (3‰) and estuarine waters (12‰), and to one hyperosmotic condition (50‰). For this, we recorded the volume of mucus exuded and compared the main stress-related biomarkers and osmosis-related parameters in skin mucus and plasma. Sea bass exuded the greatest volume of skin mucus with the highest total contents of cortisol, glucose, and protein under hypersalinity. This indicates an exacerbated acute stress response with possible energy losses if the condition is sustained over time. Under hyposalinity, the response depended on the magnitude of the osmotic change: shifting to 3‰ was an extreme salinity change, which affected fish aerobic metabolism by acutely modifying lactate exudation. All these data enhance the current scarce knowledge of skin mucus as a target through which to study environmental changes and fish status.

## 1. Introduction

European sea bass (*Dicentrarchus labrax*) is an euryhaline diadromous marine teleost species of considerable economic interest for aquaculture. Sea bass can move seasonally between seawater (SW) and fresh water (FW), and sometimes inhabit areas with fluctuating salinities such as estuaries, lagoons or coastal areas that are used as nurseries [1,2,3]. This species is also found in upper-river FW reaches [4,5]. Therefore, it is a good candidate for sea, land-based or estuarine farming. Movements from SW to FW and vice versa are usually reported for migratory diadromous species [6], while euryhaline teleost species undergo a crisis-and-regulation pattern when subjected to salinity challenges. Classically, this pattern consists of an initial phase of blood metabolic and osmotic changes, mainly related to the variation of plasma glucose, triglyceride, cholesterol, and sodium concentrations and of osmolality. This is followed by a regulation phase, which usually tends towards a steady phase [7,8,9,10]. Varsamos et al. [11] analysed the acute effects on plasma osmolality of a hypersaline environment (from a basal 35‰ to 50‰, 70‰, or 90‰) for a short period (up to 10 days). Those authors reported that plasma osmolality increased in direct relation to the intensity of the osmotic shock over the first few hours. However, 4.5 h post-challenge, plasma osmolality started to decrease to control levels, except for the 90‰ group, for which full mortality was recorded after 2.5 h. Additionally, the hyperosmotic conditions also resulted in higher drinking rates in sea bass larvae [8], which is one of the factors that regulates blood osmolality as a short-term adjustment mechanism to cope with rapid salinity changes. Laiz-Carrión et al. [12] exposed gilthead sea bream to a short-term (from 2 h to eight days) salinity challenge (from a basal 38‰ to 5‰, 15‰, and 60‰). The acute challenge (at 2 h) plasma osmolality showed a variation that agreed with the direction of the osmotic challenge: decreasing in hypoosmotic conditions and increasing in the hyperosmotic condition.

Overall, plasma cortisol values are the blood parameter that is most commonly used to indicate a stress response, irrespective of the stressor studied [13]. Although most fish respond to stress similarly, by increasing glucose, lactate, and cortisol concentrations, the response is species specific in terms of pattern and magnitude, as well as of stress tolerance [14,15,16,17,18,19,20,21]. This specificity is not limited to the species, as it also occurs between stocks or strains of the same species, and there could even be variety between individuals [20,22,23,24]. Several studies have measured the effects of an acute salinity stress on plasma biomarkers. Plasma cortisol increases in the first 2 h post-stress and returns to basal levels over the following days (4–8 days) [9,10,12,25,26,27]. In gilthead sea bream, Laiz-Carrión et al. [12] reported a tendency for glucose and lactate to increase in extreme conditions, 5‰ and 60‰, with respect to the control (38‰). However, an absence of change in glucose levels during salinity challenges has also been reported, but mostly in long-term studies [10,25]. In addition, it has been observed that plasma protein only varied when fish were transferred to hyperosmotic conditions [9,12,26].

Although several experiments have studied the effects of the osmotic challenge in European sea bass, mainly on plasma and regulatory parameters, no studies have yet considered these effects on skin mucus, a conservative indicator that can be assessed non-invasively and a potential target for stress studies [28]. Despite blood analysis generally being a non-lethal method to measure stress, the required procedure can generate injuries to fish skin and flesh, which may increase the risk of infection. Thus, alternative methods to ascertain fish stress should be considered, such as fish skin mucus analysis, which has already been demonstrated to be a reliable tool that can be used to gauge fish physiological status and well-being [17,18,28,29]. It has been reported that both endogenous and exogenous factors, such as fish developmental stage, sex, stress, infections, nutritional status, or environmental changes, can modify fish skin mucus composition [17,18,28,29,30,31,32,33,34,35]. Recently, it has been observed that the components of exuded mucus are also modified in response to stressors [36,37,38,39,40]. Some of the stress indicators, such as cortisol, glucose and lactate, have also been proposed as feasible biomarkers that can be measured in skin mucus samples [17,18,28]. Moreover, Fernández-Alacid et al. [17] demonstrated in meagre (*Argyrosomus regius*) that correlations exist between plasma and mucus for some of these indicators, in response to different acute stressors such as hypoxia and netting.

Knowledge of how sea bass respond to osmotic challenges is currently mainly related to their plasma and tissue metabolic and osmotic responses [7,8,11,22,27,41,42,43,44,45,46]. However, to date, no researchers have considered skin mucus as a target for the study of osmotic response in sea bass. Given these considerations, our main aim here was to study mucus composition during the response of juvenile sea bass to acute osmotic challenges. To this end, we transferred fish directly to two hyposaline environments, a mid-estuary condition (from a basal 35‰ to 12‰), which is practically isoosmotic with the fish internal milieu, and an almost FW condition (from 35‰ to 3‰), which is highly hypoosmotic, and also to a hypersaline condition (from 35‰ to 50‰), which is highly hyperosmotic. We explored the utility of mucus as an indicator of physiological responses during this process by evaluating the sea bass response to these osmotic challenges and measuring, for the first time, the volume of mucus exuded. In this first approach, we selected the acute response (at 3 h post-challenge) and determined the biomarker composition of the mucus, and the main stress-related biomarkers in both plasma and mucus, together with osmolality and the principal ion compositions. All our findings contribute to knowledge of the sea bass response to environmental salinities by an evaluation of skin mucus, which could be useful for conservation biology studies and aquaculture conditions.

## 2. Material & Methods

### 2.1. Animals and Experimental Procedures

European sea bass juveniles were obtained from a commercial source (Maricos de Esteros SA, Spain) and acclimated indoors at the Center of Marine Sciences (CCMAR) Ramalhete marine station (Faro, Portugal). There, they were reared for two months in open flow 1000 L fiberglass tanks supplied with running SW pumped from the marine environment, under natural temperature (15.7 ± 0.2 °C) and salinity (34.9‰ ± 0.1‰) conditions. They were exposed to a simulated natural photoperiod (April) and fed twice a day (2.5% *w*/*w*) with a commercial diet. To induce an acute osmotic challenge, closed-circuit experimental tanks (500 L) were prepared with the following nominal salinities: 3‰ and 12‰, by mixing SW with well FW, and 35‰ and 50‰, by adding the adequate amount of commercial aquarium complete sea salt (Tropic Marin, Germany). For the assay, fish (129.2 ± 3.6 g) were rapidly caught from the rearing tanks and transferred to experimental tanks, 10 fish per condition (3‰, 12‰, 35‰, and 50‰) where they were kept for 3 h. This short 3 h exposure time was selected in accordance with reported maximum effects of osmotic challenges on plasma for sea bass [22,27].

After the 3 h salinity challenge, the animals were rapidly anaesthetised with an overdose of 2-phenoxyethanol (1:250, Sigma-Aldrich, Castellón de la Plana, Spain). Individual skin mucus samples were immediately collected as described in Fernández-Alacid et al. [28] with slight modifications to obtain lateral pictures of the area from which the mucus was extracted. Briefly, fish were lightly anaesthetized with 2-phenoxyethanol (0.01%, Sigma-Aldrich, Castellón de la Plana, Spain) to avoid the stress of manipulation. Immediately, anaesthetized fish were dripped for the excess water from the tail and slightly supported on an absorbent cloth to remove ventral water excess. Then, dorsal mucus from both sides was carefully collected with a sterile glass. The sterile glass slide was gently slid along both sides of the animal only three times, to minimize epithelial cell contamination, avoiding the operculum, and both the ventral-anal and caudal fin areas. The skin mucus was then carefully pushed into a sterile tube (1.5 mL) and stored at −80 °C until analysis. Thereafter, each fish was laterally photographed (all on the left side) with a Nikon D3000 camera (Nikon, Tokyo, Japan), weighed, and measured. Blood was subsequently obtained from the caudal vein with a 1 mL heparinised syringe fitted with a 23G needle. Plasma was separated by centrifugation of whole blood at 10,000× *g* for 5 min, aliquoted, immediately frozen, and stored at −80 °C. The animals were then killed by severing the spinal cord.

The research was approved by the Centre for Marine Sciences (CCMAR)-Universidade do Algarve animal welfare body (ORBEA) and Direção-Geral de Alimentação e Veterinária (DGAV), Permit 2019-06-04-009758, in accordance with the requirements imposed by Directive 2010/63/EU of the European Parliament and of the Council of 22 September 2010 on the protection of animals used for scientific purposes.

### 2.2. Stress Biomarkers

Mucus and plasma were analysed for the stress-related biomarkers such as glucose, lactate and cortisol [17,18]. Soluble components of the skin mucus samples were obtained from the homogenised mucus, using a sterile Teflon pestle and centrifugation at 14,000× *g* as described in Fernández-Alacid et al. [28]. Enzymatic colorimetric tests (LO-POD glucose and LO-POD lactate, SPINREACT, Spain) adapted to 96-well microplates were used to measure skin mucus, and plasma glucose and lactate concentrations. Following the manufacturer’s instructions, the mucus and plasma samples, and the standard dilutions were mixed in triplicate with working reagents. The OD was determined at 505 nm with a microplate reader (Infinity Pro200 spectrophotometer, Tecan, Spain). The glucose and lactate values were expressed as mg·dL^−1^ for plasma and μg·mL^−1^ for skin mucus. Cortisol levels were measured using an ELISA kit (IBL International, Hamburg, Germany). Briefly, an unknown amount of antigen present in the sample competed with a fixed amount of enzyme-labelled antigen for the binding sites of the antibodies coated onto the wells. After incubation, the wells were washed to stop the competition reaction. Therefore, after the substrate reaction, the intensity of the colour was inversely proportional to the amount of antigen in the sample. Following the manufacturer’s instructions and adaptations for fish mucus and plasma [17,18], the samples and standard dilutions (from 0 to 3 μg·dL^−1^) were mixed with the enzyme conjugate and incubated for 2 h at room temperature. The substrate solution was added after rinsing the wells with a wash solution and incubated for 30 min. The reaction was stopped by adding stop solution and the OD was determined at 450 nm with a microplate reader (Infinity Pro200 spectrophotometer, Tecan, Spain). The cortisol values were expressed as ng cortisol mL^−1^ of plasma or skin mucus.

During the collection process, the mucus samples may have been affected by water diluting them. Thus, normalization of data through mucus protein concentration is recommendable [28] and all data from stress biomarkers are also expressed per mg of protein.

### 2.3. Total Protein Quantification

Plasma protein concentrations and skin mucus soluble protein were determined using the Bradford assay (Bradford, 1976) with bovine serum albumin (BSA) as the standard. The Bradford reagent was mixed with the samples in triplicated and incubated for 5 min at room temperature. The OD was determined at 596 nm with a microplate reader (Infinity Pro200 spectrophotometer, Tecan, Spain). The protein values were expressed as mg protein mL^−1^ of plasma or skin mucus.

### 2.4. Osmolality and Ion Quantifications of Plasma and Skin Mucus

Plasma osmolality was measured with a vapour pressure osmometer (WESCOR VAPRO 5520, Wescor Inc., Logan, UT, USA) and was expressed as mOsm·kg^−1^. Plasma Na^+^ and K^+^ levels were measured using a Flame Photometer (BWB XP, BWB Technologies, Newbury, UK) and were expressed as mmol·L^−1^. Plasma chloride concentration was measured using a colorimetric test (SPINREACT, Spain) adapted to microplates and OD was determined in a microplate reader (MultiScan Go, ThermoFisher Scientific, Tokyo, Japan). Values were expressed as mmol·L^−1^. Mucus osmolality and ion concentrations (Na^+^, K^+^ and Cl^−^) were measured using an ion analyser (ISElyte X9, Tecil, Spain). Osmolality values were expressed as mOsm·kg^−1^ and ion concentrations as mmol·L^−1^.

### 2.5. Mucus Exudation Values

To determine the effects of the osmotic challenges, total mucus exudation was obtained by measuring the volume of mucus collected (in μL) and this was related to both the skin area (in cm^2^) and fish weight (in g). For this purpose, the skin area was obtained using the ImageJ program (US National Institutes of Health, Bethesda, MD, USA). The area was manually marked as an approximation to area actually scrapped, avoiding the dorsal and the lateral fins, and over the lateral line. This was then measured using the own software for the program. Furthermore, for the first time in fish, soluble mucus collected (μL) was referred to the sampling area and to fish weight, to calculate mucus collected per area (μL·cm^−2^) and mucus collected per fish weight (μL·g^−1^).

### 2.6. Statistical Analysis

To compare the data obtained for stress-related biomarkers and osmotic parameters among the different salinity challenges, we used one-way ANOVA. Additionally, Student’s t-test was used to compare osmotic parameters between plasma and mucus. For all our statistical analysis, a prior study for homogeneity of variance was performed using Levene’s test. When homogeneity existed, Turkey’s test was applied, whereas if homogeneity did not exist, then the T3-Dunnet test was applied. Moreover, Pearson’s correlation coefficient was applied to the data to examine the relationship between plasma and mucus stress indicators. Correlations with *p* < 0.05 were considered demonstrated. Principal component analysis (PCA) was performed to study the structure of the different mucus biomarkers analysed. The PCA score plots display the main trends in the data, and their respective “weighing” reveals variables with a significant loading. All statistical analysis was undertaken using SPSS Statistics for Windows, Version 22.0 (IBM Corp, Armonk, NY, USA) and all differences were considered statistically significant at *p* < 0.05.

## 3. Results

### 3.1. Mucus and Plasma Biomarkers

Total volume of skin mucus exuded (in μL) as well as mucus exuded per unit of collection area and per unit of body weight are shown in Table 1, together with the stress-related biomarkers, such as glucose, lactate, and cortisol. Compared to mucus exuded at 35‰, a hypoosmotic shock at 3‰ or 12‰ provoked a 20% reduction in the amount of mucus collected, which was significant at the lowest salinity (150 ± 2 to 122 ± 9 µL of mucus collected, from 35‰ to 3‰, respectively, *p* < 0.05). In contrast, the acute response to the hyperosmotic shock at 50‰ caused skin mucus over-exudation: significantly 75% higher (267 ± 33 µL, *p* < 0.05) with respect to control values of fish transferred to 35‰. Mucus collected per unit of body weight followed the same significant differences as the absolute amount of mucus collected. However, no significant differences were observed when analysing the mucus per surface area of collection between control and hypoosmotic conditions. The expressions of the exuded mucus per unit of skin surface or body weight were conserved, with slight modifications, with respect to the data for total volume.

Mucus biomarkers related to stress (glucose, lactate, and cortisol) showed different responses depending on the osmotic challenges. The acute shock from 35‰ to 12‰ significantly increased mucus lactate around 3-fold (from 9.2 ± 0.8 to 25.0 ± 7.8 µg per mL), whereas it only provoked a non-significant increment of glucose of around 30%. In contrast, the stronger hypoosmotic challenge, reduced to 3‰, resulted in far lower levels of exuded lactate, and reduced to one-third the 35‰ level and less than one-seventh the 12‰ level. Consequently, the glucose/lactate ratio, an indicator of aerobic rate, was five-fold higher at 3‰. Cortisol, as the main indicator of acute stress response, was not exuded differently under acute exposure to 12‰, but at 3‰, mucus cortisol levels increased significantly by two-fold. The amounts of soluble mucus, although not directly related to the stress response, were also quantified to evaluate the possible impact on other mucus properties. In response to 3 h osmotic challenges, only the fish subjected to 3‰ showed a significant increase of mucus-soluble protein. All these biomarkers indicate a different response to the 12‰ and 3‰ challenges.

**Figure 1:**
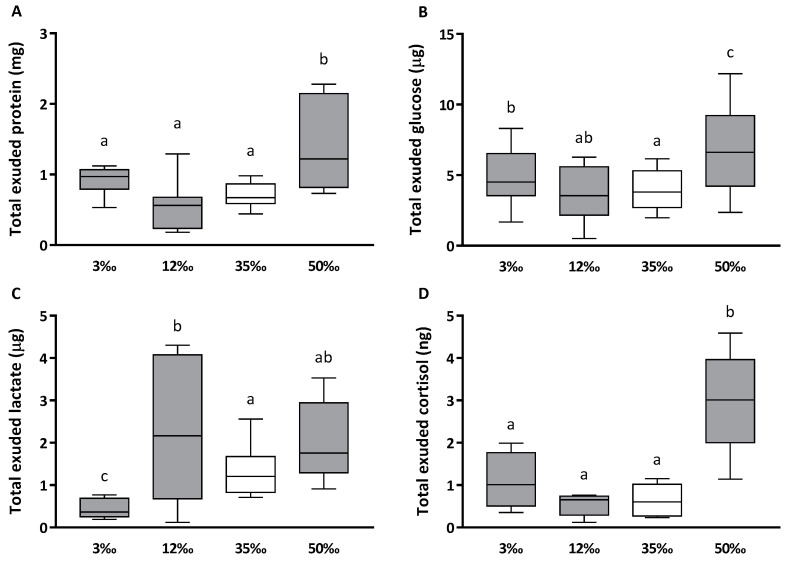
Total biomarkers exuded in skin mucus from European sea bass juveniles in response to an acute osmotic challenge. Total exuded protein (**A**), glucose (**B**), lactate (**C**) and cortisol (**D**). Values are shown as mean ± standard deviation, of ten individual samples. Different letters indicate different groups of significance among the salinity challenges (3‰, 12‰, 35‰ and 50‰) by one-way ANOVA and Tukey’s post-hoc test (*p* < 0.05). 35‰ is taken as the seawater control salinity and is represented in white.

In response to hyperosmotic shock (increased to 50‰), whereas mucus glucose, lactate and soluble protein, expressed per mL of collected mucus, did not change significantly, mucus cortisol increased significantly 2–3 folds, with respect to the 35‰ value (from 4.3 ± 1.0 to 11.5 ± 0.5 ng per mL, *p* < 0.05). Additionally, as the individual volume of mucus exuded were recorded, the total amount of each exuded biomarker in mucus are estimated and represented in Figure 1. The hypoosmotic conditions seemed to preserve nutrients, maintaining or reducing loss into mucus. Total glucose was only slightly higher in the 3‰ condition and lactate was over-secreted in the 12‰ condition, with respect to mucus values at 35‰. However, the hyperosmotic condition generated a large and significantly higher exudation of protein, glucose, and cortisol than the other conditions, in only three hours of salinity exposition, due to the greater volume of skin mucus exuded.

Plasma stress-related biomarkers 3 h post-challenge are shown in Table 2, as is the correlation with skin mucus values. No significant differences in response to acute osmotic challenges were detected for glucose and protein. Interestingly, plasma lactate showed the same pattern as observed in mucus for hypoosmotic conditions, with the lactate levels for the 3‰ condition significantly lower than control, and levels for 12‰ significantly higher. Plasma cortisol showed high values in all cases: between 300 and 700 ng per mL without any differences between conditions, possibly due to the considerable dispersion of values for this parameter. However, the lowest values were recorded for the 3‰ condition, and the highest for 12‰ and 50‰. Pearson’s correlations with mucus and plasma values only showed positive and significant correlation with lactate levels, with an r-value of 0.69 (*p* < 0.05).

### 3.2. Osmotic Parameters

**Figure 2:**
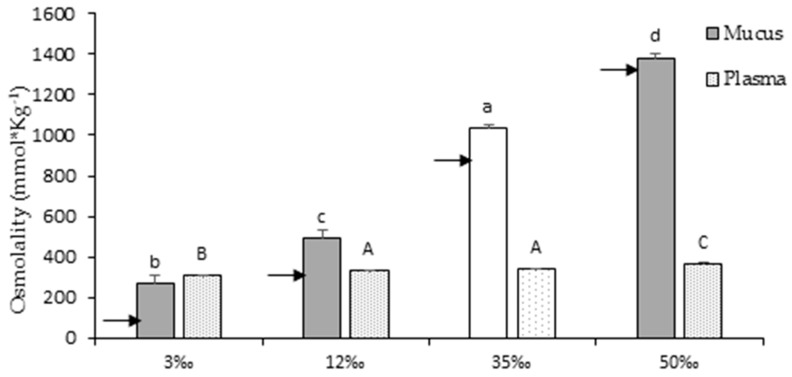
Mucus and plasma osmolality of European sea bass juveniles after 3 h of osmotic challenges. Values are shown as mean ± standard error of mean, of ten individual samples. Arrows indicate measured osmotic value of surrounding water at 3‰ = 115 mmol·kg^−1^, at 12‰ = 320 mmol·kg^−1^, at 35‰ = 931 mmol·kg^−1^, and at 50‰ = 1366 mmol·kg^−1^. Different letters indicate different groups of significance among the salinity challenges (3‰, 12‰, 35‰ and 50‰) by one-way ANOVA and Tukey’s post-hoc test (*p* < 0.05). Lower-case letters represent significant differences in mucus. Upper-case letters represent significant differences in plasma. 35‰ is taken as the seawater control salinity and is represented in white for mucus and lightly dotted for plasma.

**Figure 3:**
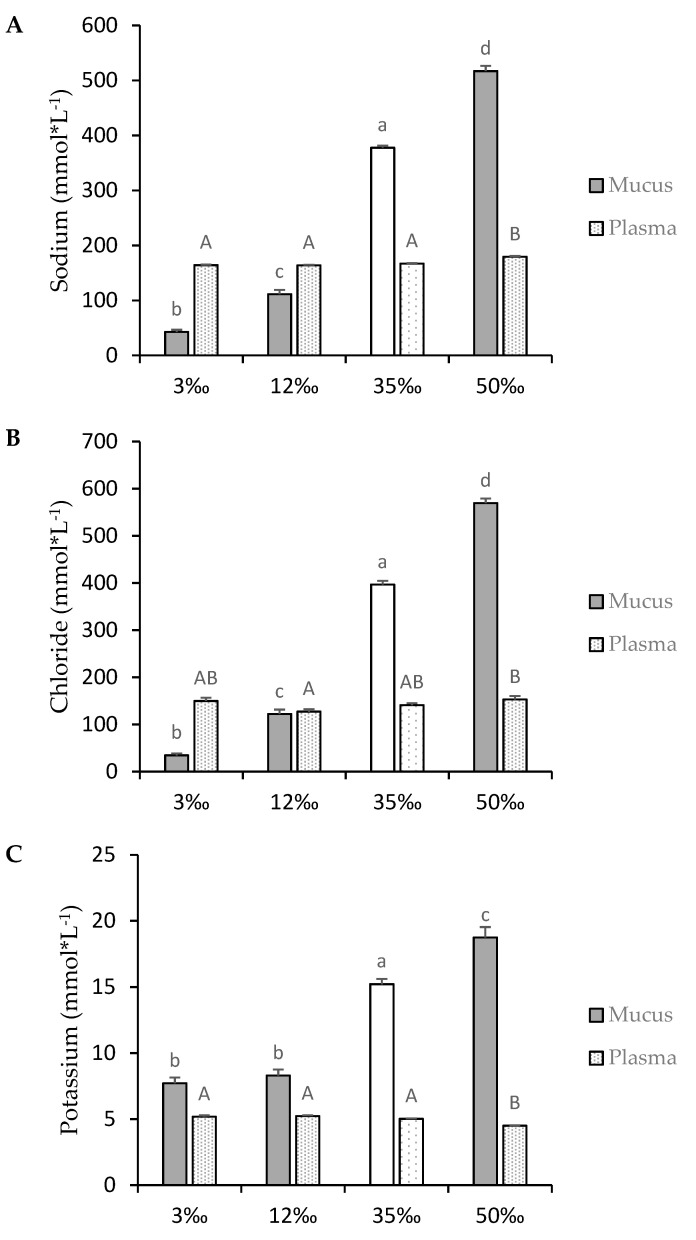
Principal mucus and plasma osmostic-related ions in European sea bass after 3 h of osmotic challenges. (**A**) Sodium (**B**) Chloride (**C**) Potassium. Values are shown as mean ± standard error of mean, of ten individual samples. Different letters indicate different groups of significance among the salinity challenges (3‰, 12‰, 35‰ and 50‰) by one-way ANOVA and Tukey’s post-hoc test (*p* < 0.05). Lower-case letters represent significant differences in mucus. Upper-case letters represent significant differences in plasma. 35‰ is taken as the seawater control salinity and is represented in white for mucus and lightly dotted for plasma.

Plasma and mucus osmolality, and the main ions concentration (Na^+^, Cl^−^, and K^+^) were measured and are shown in Figure 2 and Figure 3, respectively. To compare ion retentions in mucus or ion concentrations in plasma, the osmolality of the surrounding water was also determined. The increment in water salinity, and the concomitant increment in osmolality, was not buffered by skin mucus (Figure 2). However, at control and lower salinities, mucus tended to accumulate or retain ions, resulting in mucus having higher osmolality than the surrounding water. With regard to the main osmosis-related ions (Figure 3), Na^+^ and Cl^−^ showed a strict dependence on the surrounding water. Whereas in the 35‰ and 50‰ conditions, the sum of mucus Na^+^ and Cl^−^ reached 74.0% ± 1.3% and 75.6% ± 4.0% of mucus osmolality, respectively, at 12‰, this sum only represented 51.0% ± 3.2% while at 3‰, it was barely 35.2% ± 2.5% of the mucus osmolality. This indicates a rapid dilution of these ions in the new hypoosmotic water, proportional to the salinity reduction. The mucus concentration of potassium, although this does not contribute greatly to total osmolality values, also depended on water salinity. However, no differences were observed between the 3‰ and 12‰ conditions, which would indicate differences in the dynamics of mucus trapping potassium between these two hypoosmotic challenges.

In contrast to mucus, plasma osmolality and ions were independent of water salinity: they were generally maintained near the 35‰ control values (339 ± 3 mmol·kg^−1^). However, plasma values at 3‰ were significantly lower (311 ± 1 mmol·kg^−1^, *p* < 0.05) and at 50‰ they were significantly higher (365 ± 5 mmol·kg^−1^, *p* < 0.05), indicating some effect of this immediate stress. In plasma, the sum of the main osmosis-related ions (sodium and chloride) represented around 90% of plasma osmolality, irrespective of the challenge condition. In the 50‰ challenge, plasma ions showed differences with respect to control values at 35‰: higher sodium and chloride values, and lower potassium values.

### 3.3. Principal Component Analysis (PCA)

**Figure 4:**
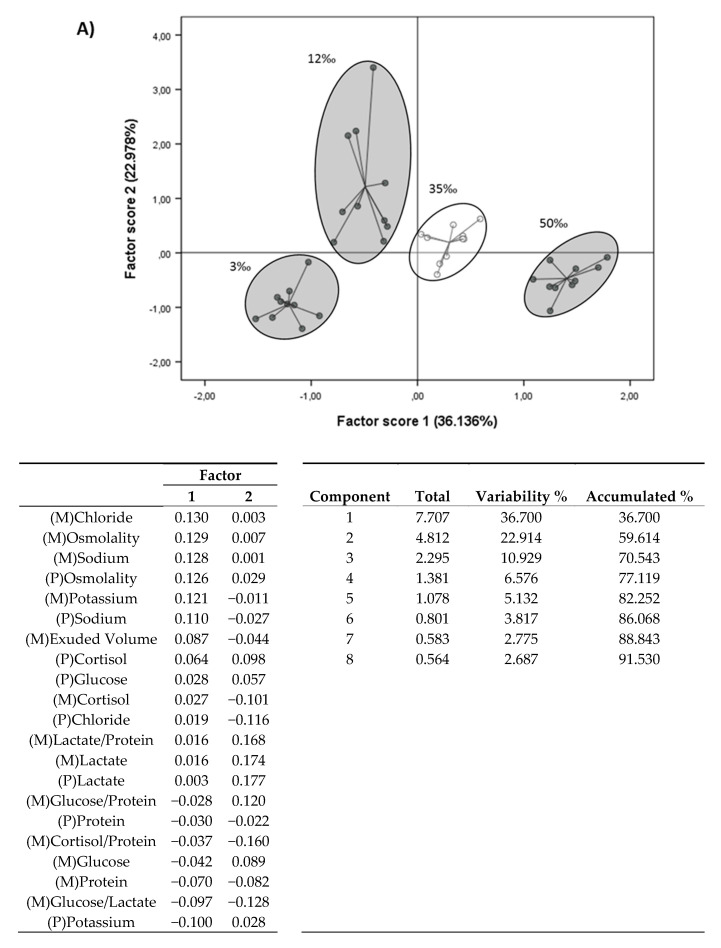

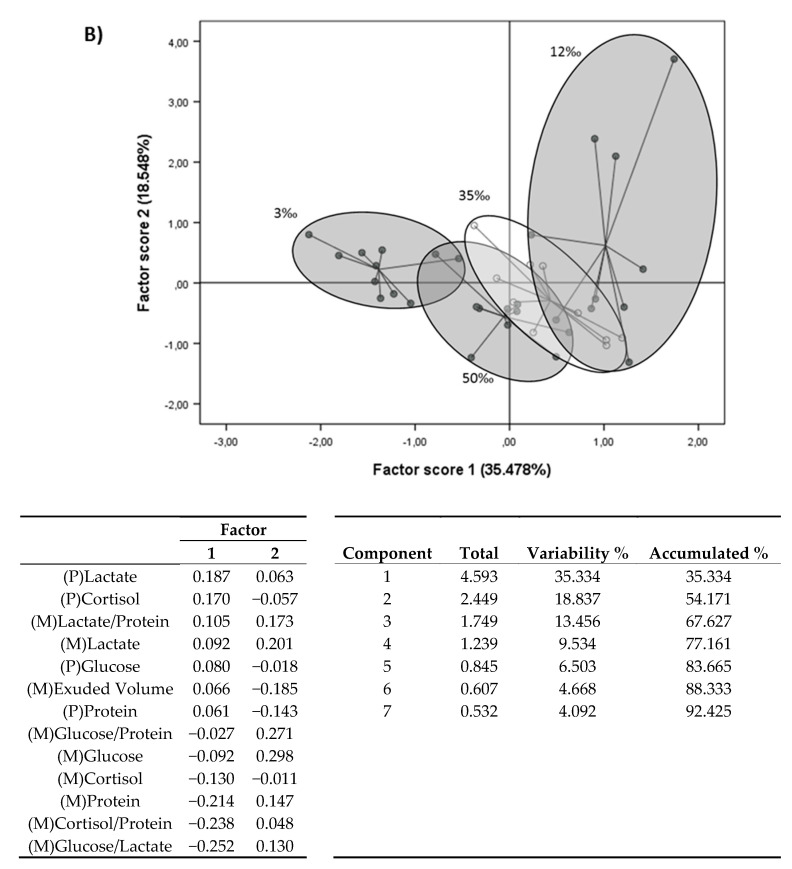
Principal component analysis (PCA) plot of European sea bass juvenile plasma and skin mucus parameters after acute osmotic stress. Factors 1 and 2 represent the first and second principal components. Parentheses indicate the variance explained by the factors. Below the figure (**A**,**B**) are tables of the contribution of the factors and the component variance accumulation, to a maximum of 90%. (**A**) PCA of plasma and skin mucus parameters including osmolality and ion parameters. (**B**) PCA of plasma and skin mucus parameters without osmolality or ion parameters.

PCA was used to determine the contribution of the stress-related and osmosis-related biomarkers to the overall response, and allowed us to discriminate the effects of the osmotic challenges over specific indicators. Figure 4 shows the PCA analysis with and without osmotic parameters. In accordance with the high impact of the surrounding water on mucus osmosis-related parameters, the PCA plot revealed the differences between challenges, clearly separating each condition on the *x*-axis: there was positive correlation with mucus chloride, osmolality and sodium, together with plasma osmolality (Factor 1 of PCA in Figure 4A). On the *y*-axis distribution, data related to lactate separated the acute response to the 12‰ and 3‰ conditions. When osmosis-related parameters were not considered (Figure 4B), in spite of a loss of confidence, the 50‰ data were close to the control values, whereas the 3‰ condition was the extreme on the *x*-axis and strongly separated from 12‰. Finally, the *y*-axis distribution showed a broad distribution of 12‰ data, probably due to the higher dispersion of values of several parameters in this condition.

## 4. Discussion

In recent years, several minimally harmful ways to evaluate fish physiological status and welfare have been tested, for instance when fish face acute biotic and abiotic stressors, including examining skin mucus. Most of the conditions in which mucus has been evaluated focus on acute stressors that occur in culture or fishery conditions, such as hypoxia, netting, crowding, anaesthetic agents or capture procedures. Research has considered different species, but mostly gilthead sea bream [28,29,34,47], rainbow trout [48], meagre [17,28,34], Senegalese sole [18] and European sea bass [28]. A few studies have reported valuable correlations between classical stress biomarkers in plasma and skin mucus, suggesting the potential to use this biological matrix instead of more invasive blood extraction [17]. Here, we assayed the stress biomarkers glucose, lactate, cortisol, and soluble protein, and some osmosis-related parameters, osmolality, and the main ions involved in plasma and skin mucus. Our aim was to determine the response of sea bass to acute (3 h) salinity challenges in two hypoosmotic conditions (3‰, diluted brackish condition; 12‰, estuarine condition) and one hyperosmotic condition (50‰), in relation to transfer to a control condition (35‰).

A volume of skin mucus is produced as one of the early response mechanisms [28,49] and should be one of the most interesting parameters to be analysed under stress conditions. To the best of our knowledge, no data on the volume of mucus collected have previously been reported in the literature. Nevertheless, some authors have reported an increase in mucus production, both when animals move from FW to SW in several migratory species, such as *Oncorhynchus nerka* [50], *Cyprinus carpio* [51], *Salmo salar* [52], *Fundus seminolis* [53] or *Colossoma macropomum* [54], and when they move from SW to FW, for *Fundulus heteroclitus* [55], *Gambusia affinis affinis*, *Catla catla* [56], and *Gasterosteus auleatus* [57], as also reviewed by Shephard [49]. In the current study, we followed our method previously described in marine fish [33] to measure the volume of mucus produced by a specific skin area surface. Thus, the exuded volume could be compared between conditions and we determined an ≈80% volume increase under the acute change from 35‰ to 50‰. In contrast, a slight, 20%, decrease was measured at 3‰ and 12‰. These data clearly indicate the different response to salinity at the skin mucosa level, and for the first time, we provide specific data for comparative purposes.

Classic indicators associated with the stress response in fish, such as glucose, lactate, and cortisol, are easily and rapidly detectable in skin mucus. During the collection process, the mucus samples may have been affected by water dilution or concentration, and in the view that environmental salinity affected mucus volume collected, it is strongly recommended to normalize data to protein levels (ratios) that proved comparable [28]. Recent studies by our group have demonstrated that a correlation exists between these parameters in plasma and mucus, such as happens on exposure to air and handling in meagre [17]. The presence of cortisol, as the main stress-related hormone, has been determined in other exocrine secretions, such as lateral line, faeces, urine, and the surrounding water, as well as in caudal fin and scales, tested in order to find a reliable non-invasive method to assess stress [47,58,59,60,61,62]. In our present study, the mucus cortisol levels indicated different stress responses depending on the osmotic challenge. Extreme conditions of 3‰ and 50‰ increased cortisol in mucus, whereas 12‰ showed similar values to control conditions. When these values are compared with plasma cortisol in order to validate mucus samples as a bioindicator, a lack of correlation was observed. In most fish species, cortisol reaches its highest concentration in plasma after 0.5–1 h, depending on the stressor and species [63,64]. However, plasma values in response to the osmotic challenges we applied here did not show significant differences with respect to control values. This fact is probably explained by the specifics of the experimental design. It must be considered that all the fish, including the control animals (35‰), were subjected to the same handling stress when transferred to the new conditions 3 h before sampling, and this probably meant that the acute osmotic effect masked the cortisol response in this short period. Measured control values were high (around 450 ng·mL^−1^) with respect to basal levels (~100 ng·mL^−1^) reported for this species (reviewed in Ellis et al., [13]). Meanwhile, the scarce data in the literature on levels of mucus exuded are still controversial. For instance, Guardiola et al. [47] found a delay between the measurement of plasma cortisol and that in skin mucus in gilthead sea bream, whereas Fanouraki et al. [27] measured the plasma cortisol to peak 1 h after stress in European sea bass. In previous studies, we observed a peak in skin mucus cortisol 1 h after air exposure stress in meagre, which strongly correlated with the plasma increment [17], while exuded cortisol did not show any post-stress dynamics in Senegalese sole [18]. As commented above, it would seem that neither skin mucus nor plasma cortisol levels are particularly informative in response to an acute osmotic challenge, at least using this experimental paradigm. This would invalidate them as mucus biomarkers. However, when considering the volume of exuded mucus (the transformation of cortisol concentration into the total amount of cortisol exuded) a marked effect of hypersalinity was detected: exuded cortisol increased five-fold with respect to control values. These data would indicate, for the first time in this species, a condition of exacerbated exudation of this hormone, which necessarily implies greater plasma release, although it was not detected. Further studies should address the cortisol dynamics, for instance, in a post-osmotic challenge time course or when subjecting fish to a sustained hypersaline condition.

An increase in skin mucus glucose and lactate exudation were widely reported after an acute stress in several fish species [17,18,22,28,48]. These responses were also reported in plasma glucose and lactate levels [12,16,17,18,25,26,48,63,65] with a strong plasma–mucus correlation reported only in meagre [17]. Fish in stressful situations exhibit increased plasma glucose as a consequence of cortisol release (reviewed in Schreck et al. [20]). However, the magnitude and duration of high glucose concentrations in plasma is species-specific [22]. Acute osmotic challenges did not alter glycaemia 3 h post-challenge comparing hypo- and hypersalinities to 35‰ values. With regard to mucus levels, to our knowledge, the only study supplying data on skin mucus glucose for similar-sized European sea bass, reported glucose values of around 10–30 µg·mL^−1^ (or 2–4 µg·mg^−1^ of protein) [28], which are in agreement with the data we present in this study. Again, when data are transformed as total glucose exudation, hypersalinity provoked the highest glucose loss via skin mucus, so sustained levels over time could be harmful for the animal. Further studies should take advantage of this mucus biomarker to evaluate the status when fish migrate from SW to FW or vice versa, as suggested for other sustained environmental conditions [28,29].

Plasma lactate increases in stressed fish, particularly if any aspect of the stressor results in increased activity or reduced oxygen availability [20,21], and such stress-related increases were also recently demonstrated in skin mucus [17,18,28,48]. Furthermore, lactate is an important metabolite that fuels osmoregulatory mechanisms [12] and should be taken into consideration, as it becomes more important during osmotic acclimation [9]. In agreement with this, our current data show that lactate was the only parameter showing a poor correlation between plasma and mucus levels. In fact, it was the only biomarker which clearly differentiated the hyposalinity conditions (3‰ and 12‰). Interestingly, whereas in the 12‰ condition both plasma and mucus lactate rose markedly within 3 h with respect to control values, in the 3‰ condition they diminished. No previous evidence exists of a direct plasma or mucus lactate reduction under hypoosmotic shock, whereas the opposite would be expected: a response similar to that occurring at 12‰ [9,25]. We could hypothesise that a more acute metabolic change would be needed in order to cope with the stress of the extreme saline condition. In view of the current results and previous studies in other species [9,12], deeper approaches are necessary to consider the related aspects with the metabolic costs, for instance histological affectations of the skin mucosa, as well as of the branchial mucosa and of the intestinal mucosa because of that the multiplying osmotic cells is certain to also have a significant metabolic cost. A change in metabolic fuel preference, by increasing lactate oxidation and stimulating the use of lactate as a gluconeogenic substrate, as was suggested for rainbow trout [66], would consume lactate faster upon its release from stores. In agreement with this, the mucus glucose/lactate ratio increased 6–7-fold in the 3‰ condition, due to the scarce lactate exuded in skin mucus. Thus, mucus lactate could be a good biomarker to measure the osmotic threshold where fish modify a classic and transient stress response to a resilient condition. Further studies are necessary to elucidate the usefulness of mucus lactate as a mucus biomarker of anaerobic/aerobic metabolic change.

Plasma osmolality has been used as a physiological indicator when measuring the effects of salinity on fish physiology [12,67,68,69,70]. Plasma osmolality is maintained between 300 and 350 mOsmol·kg^−1^ in the face of tolerable salinities by adult euryhaline teleost [12,71,72]. In our experiment, although significant differences were found between conditions, all the plasma osmolality values were in the range of 300 to 350 mOsmol·kg^−1^. These results are in agreement with those observed for gilthead sea bream after a short exposure to a salinity challenge [12]. In that previous research, the authors reported that plasma osmolality increased when fish were transferred to a 60‰ condition for the first 4 h, and it decreased when transferred to 5‰ or 15‰ for the first 24 h, achieving a steady state after four days.

Remarkably, and for the first time, skin mucus osmolality was measured during a salinity challenge, revealing that skin mucus does not completely buffer water osmolality. While at lower salinities, skin mucus tended to accumulate or retain ions, resulting in a higher osmolality than that of the surrounding water (similar to previous observations in salmonids by Roberts and Powell [73]), at higher salinities, mucus ion composition and osmolality closely reflect those of the surrounding water. Mucus substrates also contribute to maintaining an elevated osmolality and provide some hydrophobic features. This would constitute a protective barrier, decreasing the local gradient across the skin. In low salinities, the osmotic pressure of the mucus layer, being similar to that of the blood, may buffer the immediate entry of water and loss of ions across the skin. However, the elevated level of ions in the mucus of fish in high salinities may contribute to the observed volume increase as the fish tends to lose water across the skin to the immediate hyperosmotic mucus layer. Again, these values are in the framework of acute challenges. Longer-term responses of skin mucus need to be elucidated in further experiments.

Information on the functions of epidermal mucus in osmoregulation is scarce. According to Shephard [49], unstirred layers of skin mucus reduced diffusional fluxes of ions and water [74], but the impermeability conferred by skin mucus would only reduce water diffusion by about 10% of overall transport. Previously, Marshall [75] used radiolabelled Na and Cl to demonstrate that mucus could only reduce the rate of solutes permeating across the epithelia by up to 15%, but suggested, as did Kirschner [76], that mucous layers may serve to concentrate cations from ion-deficient environments and support active uptake of ions. Interestingly, the measurement of the main osmosis-related ions (sodium, chloride, and potassium) in our samples indicated rapid dilution in the new hypoosmotic water, proportional to salinity reduction. However, maintenance of ion concentrations at a basal level above environmental levels in low salinity may indicate that mucus composition is involved in or controlled by some osmoregulation process or ion capture mechanism, as suggested by Marshall [75] and Kirschner [76]. Skin mucus is a polyanionic gel [77], which increases its potential to trap cations and allow anion diffusion [78]. It remains to be seen if ion-binding proteins are secreted into the mucus under salinity challenges.

Handy [79] found higher mobility of chloride, followed by potassium and sodium, in rainbow trout skin mucus and hypothesised that most of the skin mucus ion content may reflect the goblet cell content before secretion. In addition, Roberts and Powell [52] measured whole body net efflux of ions when transferring Atlantic salmon to FW, finding a net whole-body efflux of chloride after 3 h. It has been reported that SW-acclimated fish have a “leaky” junction between gill cells that allows an efflux of ions to the environment when exposed to lower water salinity [52]. Further studies are necessary to improve our knowledge of skin mucus dynamics when fish are subjected to salinity modifications, and whether skin and gill mucus, covering the main ion exchange sites, have comparable compositions. Taking all these data into consideration, mucus osmotic modifications seem to be a good means to analyse fish responses to osmotic challenges. Moreover, when we performed PCA, the osmolality and osmotic parameters clearly discriminated between salinity groups. Components were discriminated in one direction by osmolality and osmosis-related parameters in skin mucus, as could be expected, and in the other by aerobic-to-anaerobic ratio in skin mucus and potassium concentrations in plasma. In addition, our factor 2 discriminated groups by metabolic fuel and on the opposite side by aerobic-to-anaerobic ratio and cortisol-to-protein ratio. Therefore, PCA clearly discriminated the two main effects of salinity in fish: a modification in short-term metabolic resources to cope with the new environmental situation, and the effect of the new environment on the non-buffered osmosis-related parameters in skin mucus.

## 5. Conclusions

Skin mucus biomarkers offer valuable information on the immediate response of fish to different acute challenges. The specific measurement of mucus volume per area has been shown to be a useful and informative parameter. Sea bass exuded the greatest volume of skin mucus under exposure to hypersalinity, with the highest total contents of cortisol, glucose, and protein. This indicates an exacerbated stress response with possible energy losses if the condition is sustained. Under exposure to hyposalinity, the response depends on the magnitude of the osmotic change, as 3‰ is an extreme salinity change, which probably affects fish aerobic metabolism. Although this study only focuses on the acute response, our data on skin mucus offer a new means by which to analyse fish responses to osmotic challenges, and it opens up interesting new questions on how skin mucus copes with salinity changes in the surrounding water.

## Figures and Tables

**Table 1:** Skin mucus exudation parameters and mucus biomarkers of European sea bass juveniles submitted to acute osmotic challenge.

**Salinity Challenge**	**3‰**		**12‰**		***35‰***		**50‰**	
Exudation parameters								
Collected Mucus (μL)	122.22 ± 8.78	^b^	120.00 ± 28.09	^ab^	*150.00 ± 1.51*	*^a^*	266.67 ± 33.33	^c^
Exuded mucus/skin (μL/cm^2^)	1.95 ± 0.16	^a^	1.73 ± 0.36	^a^	*2.68 ± 0.28*	*^a^*	4.82 ± 0.74	^b^
Exuded mucus/bw (μL/g)	0.93 ± 0.08	^b^	0.83 ± 0.17	^ab^	*1.28 ± 0.13*	*^a^*	2.31 ± 0.35	^c^
**Salinity Challenge**	**3‰**		**12‰**		***35‰***		**50‰**	
Mucus biomarkers								
Glucose (μg/mL)	35.31 ± 3.81	^ab^	41.65 ± 7.18	^b^	*28.53 ± 4.19*	*^ab^*	25.35 ± 2.58	^a^
Lactate (μg/mL)	3.33 ± 0.55	^a^	25.03 ± 7.84	^b^	*9.17 ± 0.84*	*^a^*	8.99 ± 1.38	^a^
Cortisol (ng/mL)	9.07 ± 2.42	^ab^	4.50 ± 0.98	^a^	*4.25 ± 1.16*	*^a^*	11.52 ± 0.54	^b^
Soluble protein (mg/mL)	6.96 ± 0.47	^b^	5.04 ± 0.44	^a^	*5.14 ± 0.37*	*^a^*	5.08 ± 0.31	^a^
Glucose/Protein (μg/mg)	5.29 ± 0.49		7.67 ± 1.24		*5.47 ± 0.63*		4.67 ± 0.46	
Lactate/Protein (μg/mg)	0.40 ± 0.04	^b^	3.45 ± 0.90	^ab^	*1.96 ± 0.19*	*^a^*	1.45 ± 0.11	^a^
Glucose/Lactate (μg/μg)	14.36 ± 1.98	^b^	2.09 ± 0.28	^a^	*3.04 ± 0.44*	*^a^*	3.05 ± 0.25	^a^
Cortisol/Protein (ng/mg)	1.40 ± 0.34	^ab^	0.85 ± 0.09	^a^	*0.83 ± 0.21*	*^a^*	2.32 ± 0.38	^b^

Values are shown as mean ± standard error of mean of ten individual samples. Different letters indicate different groups of significance among salinities challenges (3‰, 12‰, 35‰ and 50‰) by one-way ANOVA analysis and post-hoc Tuckey’s test (*p* < 0.05). 35‰ is assumed as control value of seawater salinity and represented in italic. bw = body weight.

**Table 2:** Plasma biomarkers of European sea bass juveniles in response to the acute osmotic challenge.

Plasma Biomarkers	Salinity Challenge								Plasma vs. Mucus ^1^	
	3‰		12‰		35‰		50‰		R Coefficient	*p*-Value
Glucose (mg/dL)	173.86 ± 17.64		186.22 ± 7.92		184.88 ± 11.23		188.22 ± 15.33		0.07	>0.05
Lactate (mg/dL)	35.41 ± 2.45 ^b^	^b^	105.51 ± 8.13	^c^	66.39 ± 7.64	^a^	39.86 ± 3.92	^b^	0.69	<0.01
Cortisol (ng/mL)	333.77 ± 101.67		615.88 ± 102.08		453.64 ± 80.99		586.66 ± 154.32		−0.05	>0.05
Protein (mg/mL)	20.70 ± 0.75		21.18 ± 1.02		21.14 ± 0.81		18.80 ± 0.95		0.07	>0.05

Values are shown as mean ± standard error of mean of ten individual samples. Different letters indicate different groups of significance among salinities challenges (3‰, 12‰, 35‰ and 50‰) by one-way ANOVA analysis and post-hoc Tuckey’s test (*p* < 0.05). 35‰ is assumed as control value of seawater salinity. ^1^ The relationship for each stress biomarker in plasma and mucus (n = 40 paired data, n = 20 paired data for cortisol) is analysed by Pearson’s correlations: the Pearson value (r) and significance level (*p*-value).
